# Endometrial Tuberculosis: Hysteroscopic Findings of a Clinical Case

**DOI:** 10.1055/s-0039-1692634

**Published:** 2019-06

**Authors:** Daniela Siqueira Prado, Lucas Félix Cardoso, Raimundo Dantas de Maria, Guilherme Machado de Santana, Israel Santos Marcelo, Marcela Sampaio Lima, Ângela Maria Silva

**Affiliations:** 1 Department of Medicine, Universidade Federal de Sergipe, Aracaju, SE, Brazil

**Keywords:** urogenital tuberculosis, endometrium, hysteroscopy, tuberculose urogenital, endométrio, histeroscopia

## Abstract

Endometrial tuberculosis is a rare diagnosis in the postmenopausal period, and it can mimic a carcinoma. The present article describes the case of a 54-year-old female patient with weight loss, abdominal pain, and ascites. An ultrasonography showed endometrial thickening, and a video hysteroscopy revealed a uterine cavity with formations with cotton aspect covering the entire endometrial surface and the tubal ostia. An anatomopathological evaluation diagnosed endometrial tuberculosis. The treatment was with a standardized therapeutic scheme (ethambutol, isoniazid, pyrazinamide and rifampicin), and the patient evolved with clinical improvement and normal uterine cavity at hysteroscopy. Considering the lack of pathognomonic hysteroscopic findings of the disorder, it is important to disclose the images of the case.

## Resumo

A tuberculose endometrial é um diagnóstico raro na pós-menopausa e pode mimetizar um carcinoma. O presente artigo descreve o caso de uma paciente de 54 anos com perda de peso, dor abdominal e ascite. A ultrassonografia mostrou espessamento endometrial, e a histeroscopia por vídeo revelou uma cavidade uterina com formações que apresentavam aspecto de algodão cobrindo toda a superfície endometrial e os óstios tubários. Uma avaliação anatomopatológica diagnosticou tuberculose endometrial. O tratamento foi com esquema terapêutico padronizado (etambutol, isoniazida, pirazinamida e rifampicina), e a paciente evoluiu com melhora clínica e cavidade uterina normal na histeroscopia. Considerando a falta de achados histeroscópicos patognomônicos do distúrbio, é importante divulgar as imagens do caso.

## Introduction

Tuberculosis is a major public health problem worldwide. About 10.4 million people developed tuberculosis in 2015.^1^ Urogenital tuberculosis is the 3^rd^ most common manifestation of extrapulmonary tuberculosis, preceded by lymph node tuberculosis and pleural tuberculosis.^2^ Clinical or subclinical urogenital tuberculosis affects between 4.7 and 10.4% of the individuals who have pulmonary tuberculosis.^3^ ^4^ Genital tuberculosis usually occurs secondary to tuberculosis in other sites (primarily, the lungs). The spread is generally through haematogenous or lymphatic routes.^5^ ^6^

The diagnosis of genital tuberculosis in the female genital tract should be considered in patients with risk factors such as personal or family history of tuberculosis, people who live or have traveled to endemic areas, people with clinical infertility, pelvic or abdominal pain, and menstrual disorders.^5^ The majority (75%) of women with genital tuberculosis are in the reproductive age (between 20 and 45 years old), and the detection of this disease in the postmenopausal phase is rare.^7^ In the female genital tract, the development of this disease occurs, most commonly, in the uterine tubes (90% of the cases). The infection may progress to the endometrium and to the ovaries. Vulvar or vaginal tuberculosis is exceedingly rare.^8^ ^9^

Endometrial tuberculosis is a rare finding, with few studies in the literature, most of them from underdeveloped countries, which have the highest prevalence of tuberculosis in the population.^9^ ^10^

Hysteroscopy, as a diagnostic technique, is an important tool in the detection of endometrial tuberculosis.^9^ ^11^ The most common findings described by the use of this technique are a thin endometrial thickness with dirty appearance, irregular and pale endometrium with whitish deposits linked to the surface, presence of intrauterine adhesions, and a small and slightly expandable uterine cavity.^8^ ^9^ ^10^ ^11^ Considering the lack of a specific hysteroscopic finding of endometrial tuberculosis and the low frequency of this disorder, particularly in the postmenopausal period, it is important to disclose the images of the described case.

## Case Description

A 54 year-old woman, married, menopause at 50 years old, with a previous history of 1 vaginal delivery and 1 abortion, was admitted to the Hospital Universitário of the Universidade Federal do Sergipe (HU-UFS, in the Portuguese acronym), presenting with low intensity abdominal pain, mainly located in the left iliac fossa, with weight loss, and no other associated symptoms. A total abdominal ultrasonography showed the presence of ascites, and a transvaginal ultrasonography showed centered and homogeneous endometrial echo with a thickness of 14.2 mm. No alteration was evidenced by a chest X-ray.

**Fig. 1:**
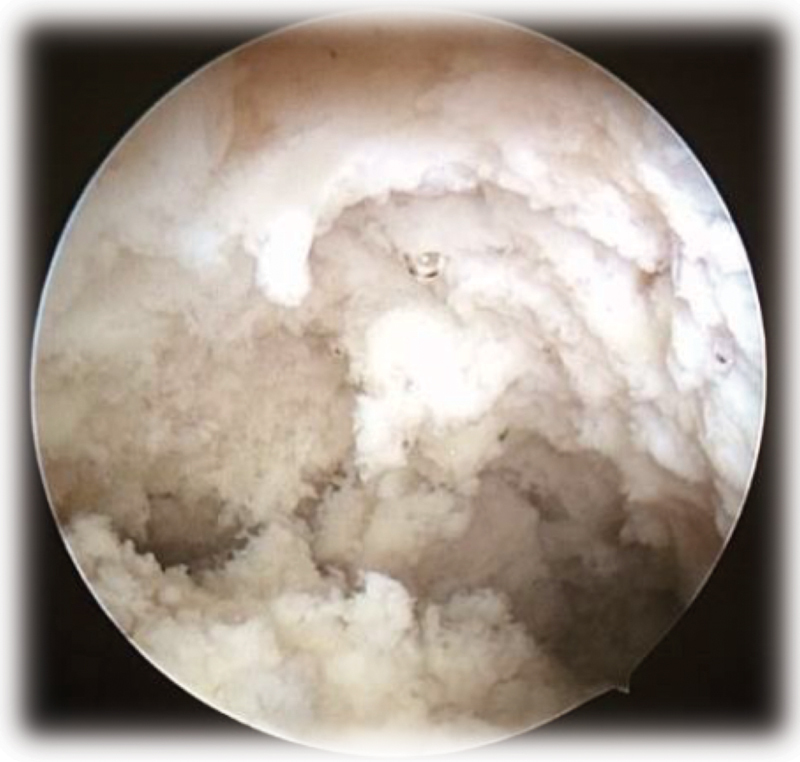
Uterine cavity with formations with cotton aspect.

**Fig. 2:**
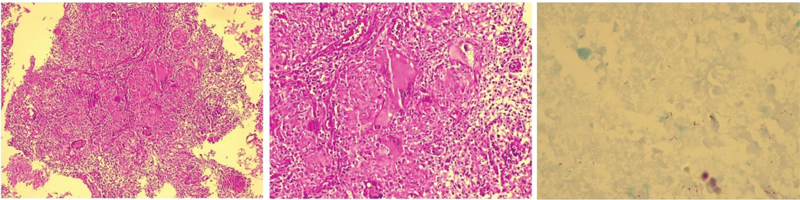
Endometrium. On the left, multiple granulomas in the endometrial stroma (hematoxylin and eosin stain, 20x). In the middle, granulomas with multinucleated giant cells (hematoxylin and eosin stain, 40x). On the right, some acid-fast bacilli in areas of caseous necrosis (Ziehl-Neelsen,100x).

A diagnostic hysteroscopy was performed and revealed a uterine cavity with formations with cotton aspect covering the entire endometrial surface and the tubal ostia, (Fig. 1) and one endocervical polyp. The endometrial biopsy showed a granulomatous endometritis with numerous epithelioid granulomas, multinucleated giant cells, and caseous necrosis (Fig. 2). There were no malignant cells in the sample. The Ziehl-Neelsen staining revealed some acid-fast bacilli in areas of caseous necrosis, confirming histological findings compatible with endometrial tuberculosis (Fig. 2). Fungus research was also performed by periodic acid-Schiff (PAS) and Grocott staining, which was negative. The cervical lesion was consistent with endocervical polyp, associated with epithelioid granulomas and multinucleated giant cells on its axis, but in a lesser degree than that observed in the endometrium. There were no atypias.

**Fig. 3:**
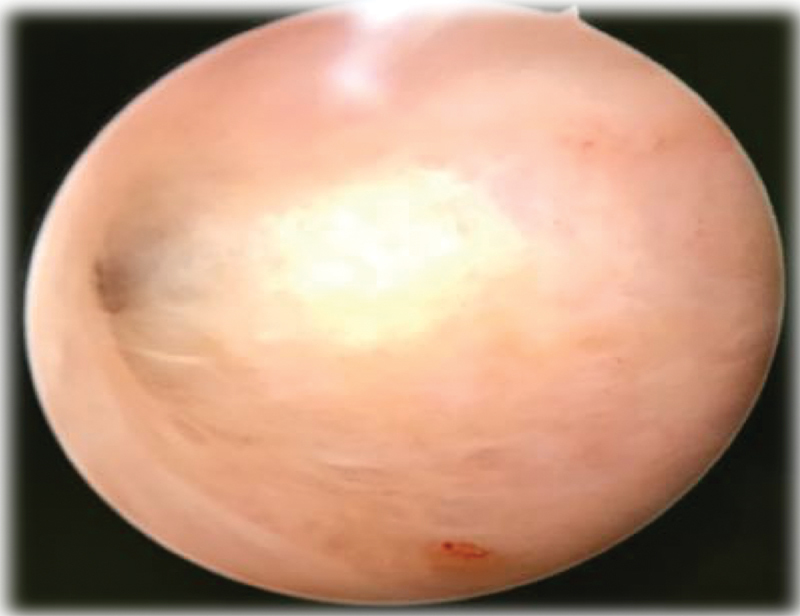
Uterine cavity after treatment.

The patient was discharged from the hospital, and was referred to the outpatient clinic of the infectology service of the HU-UFS, being treated by a standardized therapeutic scheme, using a combined fixed dose consisting of the following drugs: rifampicin (150mg), isoniazid (75mg), pyrazinamide (400mg), and ethambutol (275mg) for 2 months, as well as rifampicin (150mg) and isoniazid (75mg) for another 4 months.^5^ ^12^ The patient evolved with pain relief, regression of the ascites, and weight gain. After the treatment, a new hysteroscopy was performed and it was verified that the uterine cavity was normal (Fig. 3). A microscopic examination revealed a residual inflammatory reaction with lymphocytes and histiocytes, and only one poorly formed granuloma below the endometrial epithelium. No necrosis or bacilli were found.

## Discussion

The clinical findings of postmenopausal endometrial tuberculosis are nonspecific and difficult to diagnose in patients with no history of pulmonary tuberculosis or risk factors. Thus, in the present case, hysteroscopy was essential for the diagnosis. In a retrospective study, with 67 cases of women in infertility research who were diagnosed with endometrial tuberculosis, the presence of whitish deposits was verified, similar to those detected in the present study.^10^ ^13^ ^14^

After menopause, a clinical sign that can occur in endometrial tuberculosis is bleeding, and, less frequently, pyometra. The important differential diagnosis in this age group is endometrial cancer.^14^ ^15^ ^16^ In a report of two cases of women with postmenopausal bleeding in Brazil, the hysteroscopy found exuberant focal endometrial thickening suggesting hyperplasia, subsequently confirming the diagnosis of tuberculosis by the anatomopathological examination.^10^ In addition to pathology, techniques such as culture of mycobacteria and polymerase chain reaction (PCR) can be used for the diagnosis.^17^ Polymerase chain reaction for tuberculosis is the most sensitive indicator for the diagnosis of urogenital tuberculosis, followed by biopsy and culture.^17^

In another report of hysteroscopies of three cases of patients with endometrial tuberculosis in India, the aspect of “starry sky” was verified in one of them, by the application of a technique that uses methylene blue. This dye is not absorbed by the caseous tuberculous deposit, but it is absorbed by the normal surrounding endometrium. Thus, the unstained caseous deposit reflects the white light and the normal endometrium remains dark blue, characterizing the “starry sky” appearance. In the other two patients, the findings were alterations with the aspect of “cobwebs” and intracavitary adhesions, in addition to whitish deposits in the endometrium of one of them.^9^ In the present study, the methylene blue technique was not used and intracavitary adhesions were not verified, but whitish deposits were found.

Based on what was found, the diversity of hysteroscopic findings is confirmed, and the whitish cotton aspect may possibly be a good marker of suspicion for postmenopausal endometrial tuberculosis.
